# Factors That Influence Parental Attitudes toward Enrollment in Type 1 Diabetes Trials

**DOI:** 10.1371/journal.pone.0044341

**Published:** 2012-08-28

**Authors:** Daniela L. Buscariollo, Mario A. Davidson, Margo Black, William E. Russell, Russell L. Rothman, Daniel J. Moore

**Affiliations:** 1 Department of Pediatrics, Ian Burr Division of Endocrinology and Diabetes, Vanderbilt University Medical Center, Nashville, Tennessee, United States of America; 2 Department of Biostatistics, Vanderbilt University Medical Center, Nashville, Tennessee, United States of America; 3 Department of Medicine, Vanderbilt University Medical Center, Nashville, Tennessee, United States of America; University of Bari, Italy

## Abstract

**Aims:**

To assess parental attitudes towards type 1 diabetes clinical trials (T1DCTs) and factors that impact willingness to enroll their children with and without diabetes.

**Methods:**

A cross-sectional survey of parents of children with type 1 diabetes was administered at an academic clinic and a diabetes educational event.

**Results:**

Survey response rate was 36%. Of 166 participating parents, 76% were aware of T1DCTs. More parents reported willingness to enroll children with diabetes (47%) than unaffected children (36%). Only 18% recalled being asked to enroll their children, and of these, 60% agreed to enroll at least some of those times. Less than 30% were comfortable with placebos. Factors predicting willingness to enroll children with diabetes included healthcare provider trust, comfort with consent by proxy, low fear of child being a “guinea pig,” and comfort with placebo. Factors predicting willingness to enroll unaffected children were provider trust, comfort with consent by proxy, comfort with placebo, and perceived ease of understanding T1DCT information.

**Conclusions:**

Parents report moderate willingness to enroll children in T1DCTs. Willingness is diminished by common trial methodologies. Although most parents recalled receiving trial-related information, significantly fewer recalled being asked to participate. Efforts to optimize effective communication around identified areas of parental concern may increase T1DCT participation.

## Introduction

Clinical trials for type 1 diabetes (T1DCTs) can generate medical knowledge and improve patient care. However, it remains challenging to rapidly and optimally enroll target patient numbers in clinical trials. Trials with fewer than required participants are less likely to detect smaller, but clinically significant treatment effects, and are a potential threat to the generalizability of results [1]. Poor enrollment also leads to delayed trial completion, and consequently, to delayed knowledge and delivery of novel treatment approaches [2].

Because most initial T1D diagnoses are made in childhood, the pediatric population is the primary target for T1DCTs. Recruitment issues may be different for children and adults, and recruitment of children to clinical trials is thought to be more difficult [3]. Low accrual rates in pediatric trials relate to doctor, parent, child, and trial factors [3], [4], [5]. Some of the challenges arise because parents must ultimately decide about trial participation on behalf of their children, adding complexity to the consent process [3], [5]. Additional data suggest that the balance of perceived benefits, risks and barriers of participation influences parents' willingness to participate [3], [5], [6], [7].

Much of the research in trial participation has been performed in cancer, an immediately life-threatening disease. Less is known about parental attitudes towards enrolling their children in trials for chronic diseases, particularly type 1 diabetes. Two studies have examined the views of children themselves toward participation in T1DCTs, and another study examined how mothers who had enrolled children in the ABIS Study, a longitudinal research screening for type 1 diabetes and other multifactorial diseases, perceived trial-related information and the informed consent process [8], [9], [10]. However, little is known about the attitudes of parents/guardians towards enrolling their affected children and their children at risk for diabetes, who are also important targets for T1DCT participation. To identify factors that influence parents' decisions to enroll an at-risk or affected child in T1DCTs, we developed and administered a survey that explores a wide range of pontential influences over parental attitudes. Enhanced understanding of perceived barriers and facilitators for enrollment may aid the design of future studies and recruitment strategies, thereby optimizing trial participation and study completion.

## Results

### Demographic and enrollment data

A total of 166 questionnaires were collected, with an overall response rate of 36%. Of these, 21 questionnaires (12.7%) were completed during Vanderbilt Diabetes Family Day while the remainder was collected from clinic visits. Sociodemographic data of the sample is summarized in Table 1. The majority of parents who participated in our survey were White (90%) females (81%). Our overall clinic population (n = 1867) from which this sample was taken consists of 60% White, 10.4% Black or African American, 1.6% Hispanic, 0.5% Asian or Pacific Islander, and 27.4% pediatric patients with unknown ethnic background. To understand how our respondents related to actual clinical trial participants at our institution, we examined the demographics of the most recent enrollees at our Clinical Center in the Type 1 Diabetes TrialNet Pathways to Prevention, a longitudinal study of at-risk relatives of children with diabetes. When we examined demographics of the most recent 102 TrialNet enrollees younger than 18 years of age at our institution, a similar 89% were White and 5% were Black or African American. Most consent forms for these individuals were signed by mothers or female guardians (89%).76% of participants were aware that T1DCTs exist and 68% were aware of trials conducted locally. 66% recalled receiving information about clinical trials, which most often came from healthcare providers. The majority deemed the information received to be completely or a great deal easy to understand, although 38% reported at least some difficulty with comprehension. Only 18% recalled having been asked specifically to enroll their child with diabetes or unaffected child into a trial. Of these, 62% and 60% agreed to enroll their child with diabetes and unaffected child, respectively, at least some of the time. Parents reported slightly higher willingness to enroll children with diabetes (WTEDC, 47%) compared to unaffected children (WTEnDC, 36%). Most parents (81%) considered their child's participation in T1DCTs to be important. 67% disagreed that there is currently sufficient enrollment in T1DCTs.

**Table 1:** Demographic data for study participants and clinic population.

	Study Participants (n = 166)	Clinic Population (n = 1,867)
Mean age (range), y	39.8 (18–58)	
Sex, No. (%)		
M	31 (19)	
F	133 (81)	
Race, No. (%)		
White	149 (90)	1,119 (60)
Black or African American	11 (7)	193 (10)
Other	5 (3)	72 (4)
Unknown	0 (0)	480 (26)
Education, No. (%)		
Grade school to high school	66 (40)	
Some college to finished college	77 (47)	
Some graduate school to finished graduate school	21 (13)	
Income, No. (%)		
<$40,000	52 (33)	
$40,000–79,999	59 (37)	
≥$80,000	48 (30)	

Table 1 shows sociodemographic data for participants at the time of questionnaire completion. Ethinic background for the overall clinic population is also shown.

### Parental Perceptions of Potential Trial Benefits

When asked about motivators for enrollment, most parents reported that potential benefit for their own child (92%) and for other children in the future (87%) were important. The opportunity to contribute to science was a motivator for 43%. 31% were motivated to enroll a child by influences of family and friends. Financial compensation and increased physician access at no additional cost were motivators for 32% and 47%, respectively.

Highly desirable benefits of trial participation included findings ways to cure (91%) and prevent (78%) diabetes, to improve diabetes control (83%), and to determine risk of diabetes-related complications (72%). Parents were relatively less interested in trials designed to prolong the “honeymoon period (55%),” and to determine the risk of diabetes in unaffected children (49%).

### Effect of parent perceptions of risks and consent on willingness to enroll

**Figure 1:**
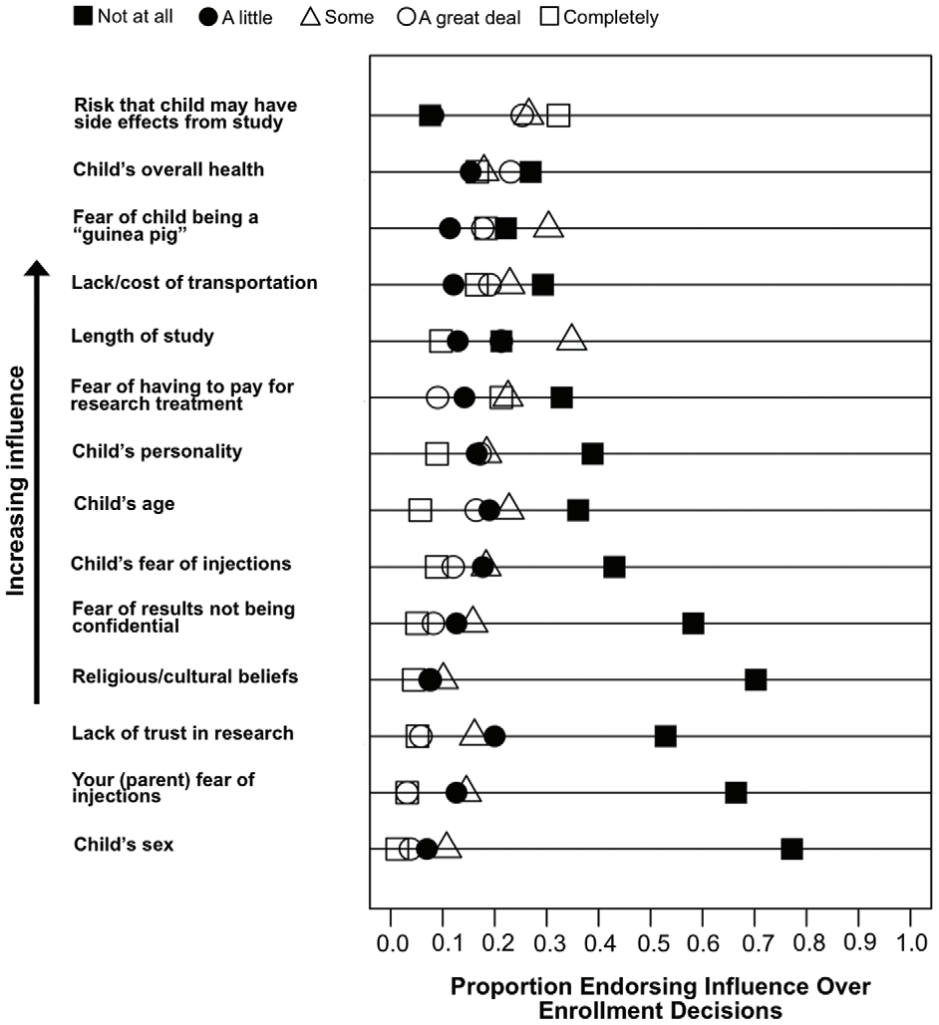
Parental responses to items probing factors that may influence decision to enroll a child in type 1 diabetes clinical trials (T1DCTs). Parents were asked: “There are lots of things that might affect a parent/guardian's decision to enroll a child in clinical trials. How much would the following influence your decision to enroll a child in a type 1 diabetes clinical trial?” Shapes correspond to endorsed degree of influence on a five-point Likert scale: black square  =  not at all, black circle  =  a little, white triangle  =  some, white circle  =  a great deal, white square  =  completely. The X-axis represents proportion of parents endorsing a specific level of influence.

A summary of factors perceived to influence parental enrollment decisions can be found in Figure 1. 57% expressed that the risk of side effects associated with trial participation has a major influence over enrollment decisions. Notably, 27% of parents endorsed discomfort with consent by proxy, or making decisions about trial enrollment on behalf of their children. Some parents were concerned about potential costs associated with trial participation –30% of parents expressed fear of having to pay for research treatment, and 36% stated that the lack or cost of transportation would influence enrollment decisions. Both fear of having to pay for research treatment and the self-described influence of transportation worries correlated with parental income (r = −0.28 and r = −0.35, respectively; p<0.01.

### Specific trial protocol elements, such as placebo and vaccines, considerably influence parental willingness

**Figure 2:**
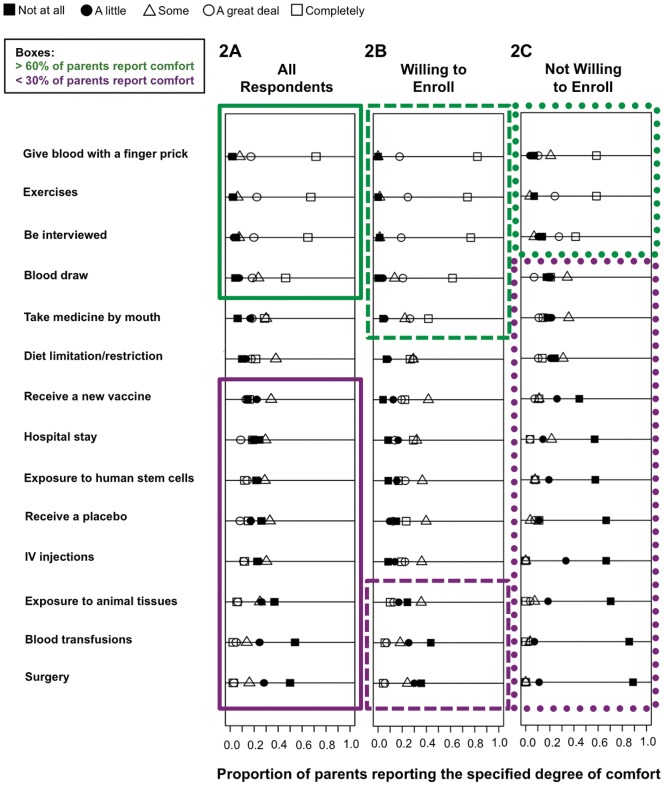
Parental responses to items probing comfort with various clinical trial protocols. Parents were asked: “We would like to know more about how specific tasks that may be part of a Type 1 diabetes clinical trial may influence your decision to enroll your child. How comfortable would you feel if your child were asked to do the following?” Shapes correspond to self-described comfort score on a five-point Likert scale: black square  =  not at all, black circle  =  a little, white triangle  =  some, white circle  =  a great deal, white square  =  completely. The X-axis represents proportion of parents endorsing a specific comfort score. “All Respondents” indicates protocol-specific comfort breakdown for all parents; “Willing to Enroll” indicates protocol-specific comfort breakdown for parents who endorsed they were “completely” or “a great deal” willing to enroll a child with diabetes; “Not Willing to Enroll” indicates protocol-specific comfort breakdown for parents who endorsed they were “not at all” or “a little” willing to enroll a child with diabetes. Breakdown for reponses depending on willingness to enroll an unaffected child are not shown as results are similar. Green box highlights tasks with which >60% of all parents were “a great deal” or “completely” comfortable. The purple box highlights tasks with which <30% of all parents were “a great deal” or “completely” comfortable. Comfort with all protocols surveyed had a statistically significant positive correlation with both willingness to enroll a child with diabetes and a non-affected child (r = 0.23–0.6). This effect can be appreciated above by the larger green and much smaller purple box among parents who reported willingness to enroll their children in T1DCTs.

Figure 2 shows attitudes towards trial participation in different scenarios reflecting potential T1DCT protocols. The majority of parents were completely or a great deal comfortable with enrolling their children in T1DCTs that involve giving blood with a finger prick, having blood drawn, and participating in exercises and interviews (Figure 2a). Conversely, over half of parents expressed they were only “a little” or “not at all” comfortable with trials requiring blood transfusions, surgery, or exposure to animal tissue and human stem cells (Figure 2a). 47% expressed comfort with trials requiring their child to take oral medications (Figure 2a).

Only 23% of parents were comfortable with the prospect of their child receiving placebo treatments (Figure 2a). As parents' income increased, so did comfort with their child receiving placebos (r = 0.21, p = 0.01). Parents who were comfortable with placebos tended to be more altruistic, as they were motivated to enroll their children by the opportunity to contribute to science and to benefit other children (r = 0.28, p<0.001 and r = 0.2, p = 0.01, respectively); however, no correlation between comfort with placebo treatment and motivation to receive benefits for one's own child was observed. Parental comfort with placebo did not correlate with lack of trust in research or with fear of child being a “guinea pig.”

Immunomodulatory interventions for type 1 diabetes involving “vaccines” are currently under investigation [11]. Yet, only 29% of parents are comfortable with enrolling their child in “vaccine” trials (Figure 2a). 19% of parents stated their decision to enroll a child in studies would be influenced by their child's fear of receiving injections (Figure 1). There was a significant negative correlation between degree of comfort with protocols involving needles and endorsement of the child's fear of needles. The presence of fear of injections in a child negatively correlated with comfort with vaccines (r = −0.42, p<0.001) and intravenous injections (r = −0.41, p<0.001).

Comfort with all protocols surveyed positively correlated with reported willingness to enroll a child with diabetes and a non-affected child (r = 0.23–0.6, p<0.01). Thus, parental comfort with the described trial elements was further broken down based on degree of parental willingness to enroll a child with diabetes in T1DCTs (Figure 2b and 2c). Breakdown for responses depending on willingness to enroll an unaffected child are not shown as results are similar. There were more protocol tasks with which greater than 60% of parents were comfortable in the group who reported willingness to enroll, as compared to parents who were less willing to enroll (Figure 2b and 2c, green boxes). Parents who were willing to enroll expressed intermediate comfort with several protocol elements, and were only resistant (less than 30% reported comfort with these tasks) to a few tasks (Figure 2b, purple box). Conversely, parents reporting unwillingness to enroll accept only the most modest protocols, and are unlikely to consider nearly all available protocols (Figure 2c, purple box), including blood draws, which is the cornestone of entry into the dominant current diabetes trial, T1D TrialNet.

### Development of a predictive model of willingness to enroll children with and without T1D

None of the sociodemographic data examined correlated with willingness to enroll, including parent age, sex, race, level of education, or income. There was a positive correlation between the perceived ease of understanding trial information and willingness to enroll (WTEDC, r = 0.25, p = 0.01; WTEnDC, r = 0.32, p<0.01).

Parents who reported greater willingness to enroll a child were less likely to be influenced by fear of side effects (WTEDC, r = −0.24, p<0.01; WTEnDC, r = −0.24, p = 0.01), and were more likely to report comfort with all trial-related protocols surveyed (Figure 2b). Willingness to enroll negatively correlated with discomfort with consent by proxy (WTEDC, r = −0.42, p<0.01; WTEnDC, r = −0.36, p<0.01).

The majority of parents (62%) agreed or strongly agreed that healthcare providers have the best interest of patients in mind when presenting options to enroll in studies. Trust in healthcare providers positively correlated with willingness to enroll one's children (WTEDC, r = 0.36, p<0.01; WTEnDC, r = 0.33, p<0.01). Moreover, few participants (11%) expressed that a lack of trust in research influences their decision of enrollment decisions. Those who reported trust in research to be an issue were less likely to be willing to enroll their children in T1DCTs (WTEDC, r = −0.28, p<0.01; WTEnDC, r = −0.26, p<0.01). 36% of parents revealed that a fear of their child being a “guinea pig” would influence their decision, which negatively correlated with willingness to enroll (WTEDC, r = −0.40, p<0.01; WTEnDC, r = −0.30, p<0.01).

As parents reporting themselves as “willing to enroll” were open to participation in substantially more protocols, we sought to define predictors of WTEDC and WTEnDC by fitting ordinal logistic models. Results are shown in Table 2.

**Table 2:** Predictors of parental willingness to enroll a child in type 1 diabetes clinical trials.

	WTEDC	WTEnDC
Discomfort with consent by proxy	0.17 (CI: 0.06–0.45) *	0.13 (CI: 0.04–0.36) *
Trust in healthcare providers	6.25 (2.17–17.97) *	5.61 (1.77–17.8) *
Trust research	1.17 (0.43–3.15)	1.08 (0.37–3.17)
Minimal fear of child being a “guinea pig”	3.52 (1.23–10.08) *	1.74 (0.59–5.14)
Fear side effects from research treatment	0.53 (0.16–1.73)	0.93 (0.26–3.36)
Comfort with placebo treatment	6.18 (CI: 2.2–17.4) *	3.98 (CI: 1.34–11.85) *
Regarded T1DCT information as only somewhat easy to understand	0.05 (CI: 0.18–1.38)	0.17 (0.05–0.52) *

WTEDC, willingness to enroll a diabetic child; WTEnDC, willingness to enroll a non-diabetic child. Results are presented as odds ratio (95% CI). *p*-values by adjusted ordinal (logistic) proportional odds models. * p<0.05.

We found that trust in healthcare providers, comfort with placebo treatment, and lack of fear of one's child serving as a “guinea pig” for research were significant positive predictors of parental WTEDC (Table 2). Discomfort with consent by proxy was a significant negative predictor of WTEDC (Table 2). Trust in research, fear of side effects associated with research participation, and perceived ease in understanding trial-related information were not statistically significant predictors (Table 2).

Although trust in healthcare providers and comfort with placebo treatment were significant positive predictors of WTEnDC, the effects were not as strong as for WTEDC (Table 2). This was similar for degree of comfort with consent by proxy (Table 2). Unlike the model for WTEDC, parents regarding T1DCT information they received as being only somewhat easy to understand were significantly less likely to be willing to enroll their unaffected children (Table 2). Results were similar for those who received no information, albeit, the effect was not as strong (odds ratio 0.4, CI: 0.17–0.97). Factors that did not predict WTEnDC include degree of trust in research, fear of side effects, and fear of child serving as a “guinea pig” (Table 2).

## Discussion

Most of the parents surveyed (81%) believed their own child's participation in T1DCTs is important. Yet, less than 50% were significantly willing to enroll their children. Although parents described themselves as being more willing to enroll their children with diabetes, for the 18% who recalled being approached about enrollment, acceptance statistics were similar for both children with and without diabetes.

While most participating parents stated they received information about T1DCTs, the majority of parents did not recall being asked specifically to enroll a child in T1DCTs. It is possible that providers believed that they asked families, but that the phrasing was not understood as a specific offer to participate. These findings suggest that if we can reach the nearly 80% of parents who may not yet have been approached, patient enrollment in T1DCTs would increase.

A notable 36% of parents reported that the information they received about trials was not entirely straightforward, which had no correlation with parental education level. Perceived degree of ease required for understanding trial information correlated with WTEDC and WTEnDC, and it was a predictor for WTEnDC. Interestingly, a previous report of a screening study for T1D demonstrated that although a majority of mothers who enrolled their children were satisfied with the information they received about the study and regarded themselves as having understood the information, a significant proportion were either unsure or disagreed with several of the basic aims of the study [10]. These findings highlights the importance of dedicating time to explain the process of trial participation, to answer questions, and to allay any misconcentions parents may have. It also suggests that development of specific adjunctive tools, such as scripts, may aid in communication and recruitment.

Although none of the sociodemographic data examined had a statistically significant correlation with willingness to enroll, financial concerns related to trial participation were of significance. A significant number of parents expressed worries of having to pay for research treatments and endorsed that lack or cost of transportation would influence enrollment decisions. Parents with lower income were more likely to endorse these concerns. These concerns may be particularly important in trials involving longer periods of time away from work and school such as trials with multi-day infusions. Therefore, when approaching parents about trial enrollment, it is worthwhile to discuss participation-related costs, perhaps even in situations where no direct costs are involved. Concerns about cost or lack of transportation also underscores the importance of coordinating research activity with regular doctor visits, and of offering any available assistance to minimize added financial and commitment burdens associated with trial participation.

Safety and comfort issues related to trial participation are of major concern for parents. Those who were more willing to enroll their children were less likely to be influenced by fear of side effects or to believe research may harm their children. Parents were less comfortable with trials involving more invasive, potentially painful protocols, or those that required tests and treatments that were not part of routine diabetes care. We found that a child's fear of receiving injections had an important influence over enrollment decisions for nearly 20% of parents. Injection fear in a child made parents significantly less comfortable with protocols that may be common aspects of T1DCTs such as blood draws, intravenous injections, vaccines, and blood transfusions. Nevertheless, intervention-specific fears may also contribute to discomfort with these protocols. Vaccines, for example, have generated considerable opposition among the public for complex reasons since their initial introduction [12].

Several studies have shown that parents generally have a poor awareness and understanding of pediatric randomized clinical trials [3], [4], [5], [13], [14], [15], [16]. Importantly, the rationale for the random allocation of treatment and the use of placebo is generally poorly understood not only by parents, but also by adult patients, adult physicians, and pediatricians [3], [4]. The implication is that the presence of randomization and a placebo group, integral aspects of high quality research, are often barriers to participation [3], [5], [17]. Results from our study agree with these reports. Only a minority of parents were comfortable with the possibility of their child being randomised to a placebo arm. Comfort with placebos had a strong positive correlation with and was predictive of willingness to enroll both a child with diabetes and an unaffected child in T1DCTs. Therefore, we need to address any existing misconceptions about placebo allocation when discussing trials with parents.

A previous study revealed that despite overall support for pediatric research, many parents are reluctant to involve their own children [5]. Although risk-benefit considerations were an important aspect of decision-making regarding trial participation [5], [7], Caldwell and workers found that many parents struggled with the responsibility of consent by proxy because of concerns about the unknown or unexpected future side effects [5]. Likewise, we observed a disconnect between parents' overall belief in the importance of trial participation and actual willingness to enroll children in studies. In our study, we find that parents' decisions regarding trial enrollment are fundamentally driven by a desire to balance potential benefits for their children with risks of side effects or potential harm from trial participation. Nevertheless, some parents were uncomfortable with the responsibility surrounding consent by proxy, which negatively impacted willingness to enroll their children. Furthermore, our results showed that discomfort with consent by proxy was a strong predictor for willingness to enroll. With regard to parents' assessment of risk-benefit, one may also hypothesize that this evaluation may be skewed immediately after diagnosis. We did not administer the survey to parents at the time of diagnosis, but many respondent families were within the first year of diagnosis. We found no correlation between willingness to enroll and the time since diagnosis. Thus, if attitudes and trial acceptance are altered immediately post-diagnosis, this effect wanes rapidly. This possible renormalization of attitudes could relate to the extensive social network of established T1D families that supports newly diagnosed families.

Our results emphasize the importance of a healthcare provider's role in the recruitment process for T1DCTs. In previous studies, parents acknowledged seeking their pediatrician's advice regarding trial participation because they trust their opinion and medical knowledge [5], [7]. Tercyak and coworkers also found that provider behavior influences adolescent acceptance of randomized clinical trials for T1D [8]. In our study, parents reported the majority of information they received about T1DCTs came from their pediatric diabetes physician. Similar to previous reports, we saw that most parents believe that doctors have the best interest of patients in mind when approaching them about trial enrollment. This belief was an important predictor of both WTEDC and WTEnDC, further highlighting the importance of the relationship between pediatricians and their patients' families in T1DCT enrollment.

Several limitations of the present study must be acknowledged. Our study population was relatively homogenous. Similar to the study reported by Caldwell et al. [5], respondents were largely white females. Likewise, we also determined that most pediatric TrialNet enrollees at our institution are White, and their consenting parents were most often mothers. Therefore, we seem to have captured the attitudes of parents with similar demographic characteristics as those who enroll their children in observational studies, but are reluctant to enter interventional trials. It is possible that mothers are the parents who most frequently bring their child to our diabetes clinic, and are thus more likely to be the ones available to fill out surveys and sign consent forms for trial enrollement. Nevertheless, fathers may influence decisions regarding a child's trial enrollment in ways that differ from that of mothers, making it important to ascertain their attitudes and to involve them in the educational and consent processes. It may also be the case that fathers and ethnic minorities are in fact just as likely to accompany their children to clinic, but are not asked as often to participate in studies. This is less likely given that the survey was made available to all parents and guardians who brought their children to our clinic. Another possibility is that fathers and ethnic minorities are less likely to be willing to participate. If fathers, minorities, or other non-responders differ in their attitudes towards T1DCTs compared to those who responded, there may be non-response bias effects. Our study was not powered to detect these differences. Although we have limited representation of responses from racial and ethnic minorities, we find it reassuring that recruitment rates for African Americans in our study (7%) are very similar to the prevalence of African American children with type 1 diabetes in the United States, according to the SEARCH for Diabetes in Youth Study (9.2%) [18]. It should also be noted that our study population is reflective of the patient population served by our institution, which may not be universally applicable.

By undertanding parents' attitudes, we can improve T1DCT recruitment, both in the sense of attaining higher enrollment rates and of making trial participation a positive experience, thereby encouraging adherence to trial protocol and participation in future studies. Although baseline willingness to enroll is not high, opinion about T1DCTs may be improved by increasing education efforts. We have identified several key modifiable parental factors concerning T1DCTs: difficulty understanding information about T1DCTs, parental concerns regarding potential costs associated with participation, discomfort with placebo treatment, and fears of their child serving as “guinea pigs” for research. Additionally, communicating more clearly and openly with parents may help address any potential conflict with consent by proxy or emotional responses to their child's illness, which has the potential to positively impact T1DCT participation.

## Materials and Methods

### Questionnaire Development

A questionnaire was designed to assess parental attitudes toward T1DCTs and to identify factors correlating with perceived willingness to enroll a child. Input from local diabetes experts was used to identify key domains expected to reflect dimensions of influence over parental attitudes. Domains identified included family sociodemographic background, child-specific characteristics, perceived risks associated with diabetes, clinical trial knowledge and experience, perceived risks and benefits associated with trial participation, perceived trial-related commitment, comfort with specific trial protocols, trust in healthcare providers and research, and motivations to enroll children in trials. The questionnaire was refined through feedback from two semistructured parent focus groups, and was piloted in 15 parents at Camp Sugar Falls (http://www.diabetes.org/living-with-diabetes/parents-and-kids/ada-camps/camps/2011/camp-sugar-falls-2011.html). The final questionnaire consisted of 48 items including open-ended, yes/no, and five-point Likert response formats (Figure S1). To assess parental willingness to enroll their children with diabetes (WTEDC) and their unaffected children (WTEnDC) in T1DCTs, parents were asked to rate their willingness to enroll each using the following scale: 1) not at all, 2) a little, 3) some, 4) a great deal, or 5) completely. Ratings of 4 and 5 were considered to represent significant willingness, and 1 and 2 ratings were considered to represent unwillingness to enroll. The protocol was approved by the Vanderbilt Institutional Review Board.

### Study Participants

Participants were parents of children with type 1 diabetes. Questionnaires were distributed at the Vanderbilt Diabetes Family Day (n = 21), a yearly gathering for pediatric diabetes patients and their families, and during routine visits at the Vanderbilt Eskind Pediatric Diabetes Clinic (VEDC) between 11/12/08–11/21/08 (n = 67) and 02/06–03/27 (n = 77). Response rate was based on number of questionnaires distributed as compared to returned at Diabetes Family Day, and the number of type 1 diabetes patients seen at the VEDC during the defined period.

To assess whether the demographic distribution our study sample adequately reflects that of our clinic population, we determined the demographic breakdown of our entire patient population. We also determined the ethnic demographics of the most recent 102 TrialNet enrollees younger than 18 years of age, and the proportion of consent forms signed by their mothers or female guardians versus fathers or male guardians.

### Data Analysis

Statistics were performed using R: A Language and Environment for Statistical Computing (R Foundation for Statistical Computing, Vienna, Austria) and significance was defined as p≤0.05. Percentages, frequencies, and Spearman's correlations were calculated to examine relationships between ordinal variables probing domains discussed above and WTEDC and WTEnDC. To determine predictors for WTEDC and WTEnDC, we fitted two adjusted ordinal (logistic) proportional odds models. Variable selection for the analysis was based on clinical relevance and potential for future intervention while refraining from overfitting the models using a limited sample size calculation based on a 10∶1 ratio of predictor to outcome events [19]. The following variables were included in the analysis for each of the two models: perception of information obtained about trials as being easy to understand, comfort with child receiving placebo treatment as part of T1DCTs, belief that providers have the interest of patients in mind when asking to enroll, belief that it should be child's decision to enroll in trials when old enough to do so (discomfort with consent by proxy), fear of child being treated as a “guinea pig,” lack of trust in research, and fear child may suffer side effects from trial participation.

## Supporting Information

Figure S1 **Parental attitudes toward T1D clinical trials survey.** The complete survey as administered to parents and analyzed in the text is shown.
(PDF)

## References

[1] Smyth RL (2001) Research with children. Paediatric practice needs better evidence – gained collaboration with parents and children. BMJ 322: 1377–1378. 11397728 10.1136/bmj.322.7299.1377 PMC1120459

[2] Walson PD (1999) Patient recruitment: US perspective. Pediatrics 104: 619–622. 10469803

[3] Caldwell PHY, Murphy SB, Butow PN, Craig JC (2004) Clinical trials in children. Lancet 364: 803–811. 15337409 10.1016/S0140-6736(04)16942-0

[4] Caldwell PHY, Butow PN, Craig JC (2002) Pediatricians' attitudes toward randomized controlled trials involving children. J Pediatr 141: 798–803. 12461496 10.1067/mpd.2002.129173

[5] Caldwell PHY, Butow PN, Craig JC (2003) Parents' attitudes to children's participation in randomized controlled trials. J Pediatr 142: 554–559. 12756389 10.1067/mpd.2003.192

[6] Tait AR, Voepel-Lewis T, Malviya S (2003) Participation of children in clinical research: factors that influence a parent's decision to consent. Anesthesiology 99. 10.1097/00000542-200310000-00012 14508312

[7] Zupancic JAF, Gillie P, Streiner DL, Watts JL, Schmidt B (1997) Determinants of parental authorization for involvement of newborn infants in clinical trials. Pediatrics 99: e6–e6. 10.1542/peds.99.1.e6 9096174

[8] Tercyak KPJ, Johnson SB, Kirkpatric KA, Silverstein JH (1998) Offering a randomized trial of intensive therapy for IDDM to adolescents. Reasons for refusal, patient characteristics, and recruiter effects. Diabetes Care 21: 213–215. 9539984 10.2337/diacare.21.2.213

[9] McGuinness C, Cain M (2007) Participation in a clinical trial: views of children and young people with diabetes. Paediatr Nurs 19: 37–39. 10.7748/paed.19.6.37.s31 17694894

[10] Stolt UG, Helgesson G, Liss P-E, Svensson T, Ludvigsson J (2004) Information and informed consent in a longitudinal screening involving children: a questionnaire survey. Eur J Hum Genet 13: 376–383. 10.1038/sj.ejhg.5201336 15657607

[11] Ludvigsson J, Hjorth M, Chéramy M, Axelsson S, Pihl M, et al (2011) Extended evaluation of the safety and efficacy of GAD treatment of children and adolescents with recent-onset type 1 diabetes: a randomised controlled trial. Diabetologia 54: 634–640–640. 21116604 10.1007/s00125-010-1988-1

[12] Poland GA, Jacobson RM (2011) The Age-Old Struggle against the Antivaccinationists. N Engl J Med 364: 97–99. 21226573 10.1056/NEJMp1010594

[13] Hartch SC, Thong YH (1995) Parental perceptions and attitudes about informed consent in clinical research involving children. Soc Sci Med 41: 1647–1651. 8746864 10.1016/0277-9536(95)00058-f

[14] Tait AR, Voepel-Lewis T, Malviya S (2003) Do they understand? (Part I): parental consent for children participating in clinical anesthesia and surgery research. Anesthesiology 98. 10.1097/00000542-200303000-00005 12606901

[15] Snowdon C, Garcia J, Elbourne D (1997) Making sense of randomization; responses of parents of critically ill babies to random allocation of treatment in a clinical trial. Soc Sci Med 45: 1337–1355. 9351153 10.1016/s0277-9536(97)00063-4

[16] Wiley F M, Ruccione K, Moore I M, McGuire-Cullen P, Fergusson J, et al (1999) Parents' perceptions of randomization in pediatric clinical trials. Children Cancer Group. Cancer Pract 7: 248–256. 10687594 10.1046/j.1523-5394.1999.75010.x

[17] Welton AJ, Vickers MR, Cooper JA, Meade TW, Marteau TM (1999) Is recruitment more difficult with a placebo arm in randomised controlled trials? A quasirandomised, interview based study. BMJ 318: 1114–1117. 10213724 10.1136/bmj.318.7191.1114 PMC27847

[18] Group SfDiYS (2006) The Burden of Diabetes Mellitus Among US Youth: Prevalence Estimates From the SEARCH for Diabetes in Youth Study. Pediatrics 118: 1510–1518. 17015542 10.1542/peds.2006-0690

[19] Harrell FJ (2001) Regression Modeling Strategies: With Applications to Linear Models, Logistic Regression, and Survival Analysis. New York: Springer.

