# Supplementary material for: Factors That Influence Parental Attitudes toward Enrollment in Type 1 Diabetes Trials
Source: PLoS One. 2012 Aug 28;7(8):e44341. doi: 10.1371/journal.pone.0044341 (PMC3429434; doi:10.1371/journal.pone.0044341)
Supplement: Figure S1 — Parental attitudes toward T1D clinical trials survey. The complete survey as administered to parents and analyzed in the text is shown. (PDF) [file pone.0044341.s001.pdf]

Supplemental Figure 1. Parental attitudes towards type 1 diabetes clinical trials survey

PERSONAL BACKGROUND

1. How far do you travel from home to get to appointments here at Vanderbilt?
   __________ minutes/ hours of driving

2. Guardian's (**your**) year of Birth: ____________

3. Guardian's (**your**) Sex:
   - ❑ Female
   - ❑ Male

4. What is your race?
   - ❑ White
   - ❑ Black or African-American
   - ❑ Asian
   - ❑ Pacific Islander
   - ❑ American Indian or Alaska Native
   - ❑ Other: _______________________

5. Guardian's (**your**) highest level of education completed:
   - ❑ Less than high school
   - ❑ High school or GED
   - ❑ 2 year college
   - ❑ 4 year college/university
   - ❑ Graduate school

6. Which one of the following categories best describes your yearly household income from all sources?
   - ❑ Less than $40,000
   - ❑ $40,000 to $59,999
   - ❑ $60,000 to $79,999
   - ❑ $80,000 to $99,999
   - ❑ $100,000 and above
   - ❑ I do not know

7. What is **your child's** current insurance coverage?
   - ❑ TennCare only
   - ❑ Medicaid only
   - ❑ Private insurance only
   - ❑ TennCare + Medicaid
   - ❑ TennCare + private insurance
   - ❑ Medicaid + private insurance
   - ❑ TennCare + Medicaid + private insurance

FAMILY HISTORY OF TYPE 1 DIABETES:

1. How many children do you have? (They can be of *any age*. Please include children by birth, by marriage, and by adoption) _____ (number of children)

2. For your child who has had Type 1 diabetes the **longest**, in what year was he/she first diagnosed? __________ (YYYY)

3. Please rank **how much you agree** with this statement: "I am worried my child will develop complications from diabetes":

☐1	☐2	☐3	☐4	☐5
(strongly disagree)	(disagree)	(neutral)	(agree)	(strongly agree)

4. If you have a child/children with Type 1 diabetes, what was his/her/their **most recent A1c**? Please choose the number that is closest to this A1c value for **<u>each child</u>**:

**Child 1:**	**Child 2:**	**Child 3:**	**Child 4:**
☐4% ☐5% ☐6% ☐7%	☐4% ☐5% ☐6% ☐7%	☐4% ☐5% ☐6% ☐7%	☐4% ☐5% ☐6% ☐7%
☐8% ☐9% ☐10% ☐11%	☐8% ☐9% ☐10%☐11%	☐8% ☐9% ☐10%☐11%	☐8% ☐9% ☐10%☐11%
☐12%☐13% ☐14%☐15%	☐12%☐13% ☐14%☐15%	☐12%☐13% ☐14%☐15%	☐12%☐13% ☐14%☐15%

HISTORY OF CLINICAL TRIAL PARTICIPATION

1. Are you aware that there are clinical trials for **Type 1 diabetes**?
   - ❑ No
   - ❑ Yes

2. Are you aware that there are clinical trials for Type 1 diabetes **at Vanderbilt**?
   - ❑ No
   - ❑ Yes

3. From what **sources** have you received information about Type 1 diabetes clinical trials (including Vanderbilt sources and all other sources)?
   - ☐ None
   - ☐ Family/Friend
   - ☐ Media (internet, TV, radio, etc)
   - ☐ Healthcare provider (Nurse, Physician, etc.)
   - ☐ Other: ____________________

4. Was the information you received about Type 1 diabetes clinical trials **easy to understand**?

☐1	☐2	☐3	☐4	☐5	☐N/A
(not at all)	(a little)	(some)	(a great deal)	(completely)	(not applicable)

5. Have you ever ***<u>been asked</u>*** to enroll a child **with Type 1 diabetes** in a Type 1 diabetes clinical trial?
   - ❑ No
   - ❑ Yes, and I agreed to enroll **every time** I was asked.
   - ❑ Yes, and I agreed to enroll **some of the times** I was asked.
   - ❑ Yes, but I chose **not** to enroll **every time** I was asked.

6. Have you ever ***<u>been asked</u>*** to enroll a child **who does not have** Type 1 diabetes in a Type 1 diabetes clinical trial (ex: a clinical trial to prevent or better understand Type 1 diabetes)?
   - ❑ Not applicable - I have no children that *do not* have Type 1 diabetes.
   - ❑ No
   - ❑ Yes, and I agreed to enroll **every time** I was asked.
   - ❑ Yes, and I agreed to enroll **some of the times** I was asked.
   - ❑ Yes, but I chose **not** to enroll **every time** I was asked.

7. If you ***have*** **been asked** to enroll **any** of your children in a Type 1 diabetes clinical trial, do you feel that your healthcare providers gave you **enough clear information** to make the decision to enroll or not to enroll your child?
   - ❑ Not applicable - I have ***not*** **been asked** to enroll any children in a Type 1 diabetes clinical trial.
   - ❑ No
   - ❑ Yes, **some** of the times.
   - ❑ Yes, **most** of the times.
   - ❑ Yes, **all** of the times.

8. If you were **now** approached about enrolling a child **with Type 1 diabetes** in a Type 1 diabetes clinical trial, how **willing** would you be to enroll him/her?

☐1	☐2	☐3	☐4	☐5
(not at all)	(a little)	(some)	(a great deal)	(completely)

9. If you were **now** approached about enrolling a **non-diabetic child** in a Type 1 diabetes clinical trial (ex: prevention trial), how **willing** would you be to enroll him/her?

☐1	☐2	☐3	☐4	☐5	☐N/A
(not at all)	(a little)	(some)	(a great deal)	(completely)	(Not Applicable)

CLINICAL TRIAL GOALS: *A clinical trial is scientific research to generate knowledge for better medical practice*. Please share with us how the goal of the clinical trial would affect your decision to enroll your child. How **interested would you be in enrolling your child/children** in studies with the following *goals:*

	Not at all	A little	Some	A great deal	Completely
1. Studies that may determine the *risk of type 1 diabetes* in my non-diabetic children.	☐1	☐2	☐3	☐4	☐5
2. Studies that may determine the *risk of complications* in my child/children with diabetes.	☐1	☐2	☐3	☐4	☐5
3. Studies that may *improve diabetes control* for my child	☐1	☐2	☐3	☐4	☐5
4. Studies that may increase the time that my child with diabetes *does not need shots* (the "honeymoon" period).	☐1	☐2	☐3	☐4	☐5
5. Studies that may *reverse* ("cure") diabetes	☐1	☐2	☐3	☐4	☐5
6. Studies that may *prevent* diabetes	☐1	☐2	☐3	☐4	☐5

INTEREST IN TYPES OF CLINICAL TRIALS

1. Each clinical trial is different, so people who participate may have to do different things depending on the study. We would like to know more about how *specific tasks* that may be part of a Type 1 diabetes clinical trial *may influence your decision* to enroll your child. **How comfortable would you feel if your child were asked to**:

	Not at all	A little	Some	A great deal	Completely
a. Give blood with a finger-prick	☐1	☐2	☐3	☐4	☐5
b. Have their blood drawn	☐1	☐2	☐3	☐4	☐5
c. Take IV injections	☐1	☐2	☐3	☐4	☐5
d. Receive blood transfusions	☐1	☐2	☐3	☐4	☐5
e. Do exercises	☐1	☐2	☐3	☐4	☐5
f. Be interviewed	☐1	☐2	☐3	☐4	☐5
g. Stay in the hospital to receive medicine	☐1	☐2	☐3	☐4	☐5
h. Have diet limited/restricted	☐1	☐2	☐3	☐4	☐5
i. Take medicine by mouth	☐1	☐2	☐3	☐4	☐5

	Not at all	A little	Some	A great deal	Completely
j. Undergo surgery	☐1	☐2	☐3	☐4	☐5
k. Be exposed to animal tissues (ex: cow or pig islets)	☐1	☐2	☐3	☐4	☐5
l. Be exposed to human stem cells	☐1	☐2	☐3	☐4	☐5
m. Receive a new vaccine	☐1	☐2	☐3	☐4	☐5
n. Receive a placebo (inactive medicine)	☐1	☐2	☐3	☐4	☐5

2. For the tasks where you marked **"not at all"** or only **"a little"** comfortable, please list any reasons why you were not as comfortable with these tasks:

____
____
____
____
____

3. There are lots of things that might **affect** a parent/guardian's decision to enroll a child in clinical trials. How much would the following **influence your decision to enroll a child** in a Type 1 diabetes clinical trial?

	Not at all	A little	Some	A great deal	Completely
a. Your religious or cultural beliefs	☐1	☐2	☐3	☐4	☐5
b. Your child's age	☐1	☐2	☐3	☐4	☐5
c. Your child's sex	☐1	☐2	☐3	☐4	☐5
d. Your child's personality	☐1	☐2	☐3	☐4	☐5
e. Your child's overall health	☐1	☐2	☐3	☐4	☐5
f. The length of the study	☐1	☐2	☐3	☐4	☐5
g. Lack/cost of transportation	☐1	☐2	☐3	☐4	☐5
h. Any fear **you have** of giving injections	☐1	☐2	☐3	☐4	☐5
i. Any fear **your child has** of receiving injections	☐1	☐2	☐3	☐4	☐5
**j.** Any fear of your child being a "guinea pig"	☐1	☐2	☐3	☐4	☐5
k. Any fear of results not being private/ confidential	☐1	☐2	☐3	☐4	☐5
l. Any fear of having to pay for the research treatment	☐1	☐2	☐3	☐4	☐5
m. Lack of trust in research	☐1	☐2	☐3	☐4	☐5
n. Risk that your child may have side-effects from the study	☐1	☐2	☐3	☐4	☐5

4. There are many things that might **motivate or encourage** a parent/guardian to enroll a child in clinical trials. How much would the following **make you want to enroll a child** in a Type 1 diabetes clinical trial?

	Not at all	A little	Some	A great deal	Completely
a. Contributing to science	☐1	☐2	☐3	☐4	☐5
b. Benefit for **your** child	☐1	☐2	☐3	☐4	☐5
c. Benefit for **other** children in the future	☐1	☐2	☐3	☐4	☐5
d. More access to your doctor at no cost as part of the study	☐1	☐2	☐3	☐4	☐5
e. Money/financial compensation	☐1	☐2	☐3	☐4	☐5
f. Influence of friends and relatives	☐1	☐2	☐3	☐4	☐5

TRIAL COMMITMENT: We would like to know more about how *the degree of commitmen*t involved in participating in Type 1 diabetes clinical trial *may influence* your decision to enroll your child. **Please tell us what you think is appropriate to ask of you and your child in the following situations:**

1. If your child were placed on an oral medicine, **how many times each day** would you be willing to administer it? …………☐0 ☐1 ☐2 ☐3 ☐4 ☐5 ☐ More times/day
2. If your child were placed on an oral medicine, **for how long** would you be willing to administer it? …………☐0 ☐6mo ☐1yr ☐2 yr ☐5 yr ☐10 yr ☐Lifetime
3. If your child were placed on an injected medicine, **how many times each day** would you be willing to administer it? ……..☐0 ☐1 ☐2 ☐3 ☐4 ☐5 ☐ More times/day
4. If your child were placed on injected medicine, **for how long** would you be willing to administer it? ………..☐0 ☐6 mo ☐1yr ☐2 yr ☐5 yr ☐10 yr ☐Lifetime
5. **How many times per year** would you be willing to allow your child's blood to be drawn? ………☐0 ☐1 ☐2 ☐3 ☐4 ☐5 ☐ More times/year
6. **How many times per year** would you be willing to come to the clinic with your child? ………☐0 ☐1 ☐2 ☐3 ☐4 ☐5 ☐ More times/year

ATTITUDES TOWARDS CLINICAL TRIALS: Please read the following statements and indicate how much you **agree** with each by scoring on a scale from 1(strongly disagree) to 5 (strongly agree)

	**Strongly disagree**	**Disagree**	**Neutral**	**Agree**	**Strongly agree**
1. My child's participation in Type 1 diabetes research is important to find a cure.	☐1	☐2	☐3	☐4	☐5
2. It is more important to give money to Type 1 diabetes research than to participate in it.	☐1	☐2	☐3	☐4	☐5
3. Enough people are already participating in Type 1 diabetes clinical trials.	☐1	☐2	☐3	☐4	☐5
4. Healthcare providers generally have the best interest of patients in mind when they ask them to enroll in clinical trials.	☐1	☐2	☐3	☐4	☐5
5. The decision to enroll my child in a clinical trial makes me uncomfortable because I feel it is a decision my child should make when he/she is old enough to do so.	☐1	☐2	☐3	☐4	☐5
6. I am afraid research may harm my child/children	☐1	☐2	☐3	☐4	☐5
7. Drug companies will prevent a cure from being tested	☐1	☐2	☐3	☐4	☐5
8. I would be uncomfortable with a trial that could cause *any* increase in the risk of diabetes in my non-diabetic children.	☐1	☐2	☐3	☐4	☐5
9. I am afraid that a diabetes prevention trial could *cause* my at-risk child to develop diabetes.	☐1	☐2	☐3	☐4	☐5
10. I would value protection from complications of diabetes almost as much as a cure.	☐1	☐2	☐3	☐4	☐5
11. If my child became uncomfortable (tummy ache, occasional nausea with medicine) taking a medicine, I would withdraw from the trial.	☐1	☐2	☐3	☐4	☐5
12. I would give my non-diabetic child one shot every day to delay diabetes for 5 years or more.	☐1	☐2	☐3	☐4	☐5

Thank you for completing our survey. Please slide it in the locked box when you are finished.

ADDITIONAL COMMENTS: ____
